# In vitro RNA release from a human rhinovirus monitored by means of a molecular beacon and chip electrophoresis

**DOI:** 10.1007/s00216-016-9459-2

**Published:** 2016-03-28

**Authors:** Victor U. Weiss, Christina Bliem, Irene Gösler, Sofiya Fedosyuk, Martin Kratzmeier, Dieter Blaas, Günter Allmaier

**Affiliations:** Institute of Chemical Technologies and Analytics, Vienna University of Technology (TU Wien), Getreidemarkt 9/164, 1060 Vienna, Austria; Department of Medical Biochemistry, Medical University of Vienna, Vienna Biocenter, Dr. Bohr-Gasse 9, 1030 Vienna, Austria; Agilent Technologies, Hewlett-Packard-Straße 8, 76337 Waldbronn, Germany

**Keywords:** Virus, Electrophoresis, Molecular beacon, Fluorescence, RNA uncoating

## Abstract

Liquid-phase electrophoresis either in the classical capillary format or miniaturized (chip CE) is a valuable tool for quality control of virus preparations and for targeting questions related to conformational changes of viruses during infection. We present an in vitro assay to follow the release of the RNA genome from a human rhinovirus (common cold virus) by using a molecular beacon (MB) and chip CE. The MB, a probe that becomes fluorescent upon hybridization to a complementary sequence, was designed to bind close to the 3′ end of the viral genome. Addition of Trolox (6-hydroxy-2,5,7,8-tetramethylchroman-2-carboxylic acid), a well-known additive for reduction of bleaching and blinking of fluorophores in fluorescence microscopy, to the background electrolyte increased the sensitivity of our chip CE set-up. Hence, a fast, sensitive and straightforward method for the detection of viral RNA is introduced. Additionally, challenges of our assay will be discussed. In particular, we found that (i) desalting of virus preparations prior to analysis increased the recorded signal and (ii) the MB–RNA complex signal decreased with the time of virus storage at −70 °C. This suggests that 3′-proximal sequences of the viral RNA, if not the whole genome, underwent degradation during storage and/or freezing and thawing. In summary, we demonstrate, for two independent virus batches, that chip electrophoresis can be used to monitor MB hybridization to RNA released upon incubation of the native virus at 56 °C.

**Electronic supplementary material:**

The online version of this article (doi:10.1007/s00216-016-9459-2) contains supplementary material, which is available to authorized users.

**Graphical Abstract:**
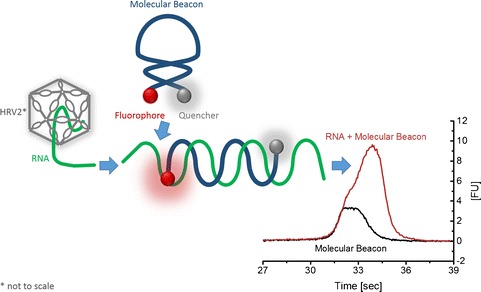
Schematic of the study strategy: RNA released from HRV-A2 is detected by chip electrophoresis through the increase in fluorescence after genom complexation to a cognate molecular beacon

## Introduction

Human rhinoviruses (HRVs) are non-enveloped, icosahedral particles of approx. 30 nm diameter [1, 2]. They are composed of four viral proteins (VP1–VP4), 60 copies each, forming the virus capsid, and a single-stranded, positive sense RNA genome of approx. 7.1 kb covalently linked to the approx. 20 amino acid residue peptide VPg at the 5′ end. The 3′ end carries a poly-(A) tail [3]. To date, more than 160 serotypes have been characterized with respect to their genome sequence. On top of the phylogenetic classification into species A, B and C, the first two have also been divided into a minor and a major receptor group [4]. Minor group viruses bind members of the low density lipoprotein receptor (LDLR) family and major group viruses bind intercellular adhesion molecule 1 (ICAM1) for cell entry. In the current work HRV serotype 2 (HRV-A2), a minor group prototype strain, was used.

Upon binding its receptor on the plasma membrane of the host cell, HRV-A2 is taken up into endosomes where the acidic pH induces structural changes culminating in the release of the viral RNA genome that is supposedly shuttled across the endosomal membrane into the cytosol in a process termed virus uncoating. There, it is translated into a polyprotein that undergoes proteolytic cleavages resulting in the structural and the non-structural proteins responsible for replication [4]. Subviral particles in which the RNA genome is prepared for release from the protective, proteinaceous viral shell can also be obtained in vitro and have been extensively characterized [5, 6]; in the process of virus uncoating native virions (composed of VP1 through VP4 and RNA/VPg and sedimenting at 150S) first release VP4 and expose amino-terminal sequences of VP1. Resulting subviral A particles composed of VP1 through VP3 and RNA/VPg sediment at 135S. In a second step, the viral RNA (with the attached VPg) is released and empty capsids (particles sedimenting at 80S) are obtained [5]. In vitro, the alteration of HRV-A2 can either be triggered by acidification (pH ≤5.8), which mimics the in vivo situation in endosomes, or by heating (≥10 min to ≥56 °C) [7]. Depending on the concentration of divalent cations, subviral A particles or empty viral capsids are formed [8, 9]. Several orthogonal analytical methods like sucrose density centrifugation (e.g. ref. [5]), transmission electron microscopy [10], nano electrospray gas-phase electrophoretic mobility molecular analysis (nES GEMMA) [2, 11] and capillary electrophoresis (CE) [7, 8, 12, 13] have been employed in their characterization. Fluorescence (FL) labelling of the virus capsid with *N*-hydroxysuccinimide-activated dyes targeting primary amines i.e. mainly lysine residues on the virus surface [14] or non-covalent labelling of the RNA genome [15] or both [16] allowed detection of virus particles with high sensitivity.

Furthermore, in a series of publications we reported the electrophoretic analysis of fluorescently labelled HRV-A2 in the chip format [17, 18]. We employed a commercially available instrument (Agilent 2100 Bioanalyzer) equipped with simultaneous FL detection at two wavelengths (λ_ex/em_ = 470/525 and 635/685 nm, respectively) and chips from soda-lime glass in the absence of any sieving material and in the presence of electroosmosis. Other than classical CE in the capillary format, chip electrophoresis allowed analysis within less than 2 min (including electrophoretic sample injection). Additionally, electrophoresis of virions in the capillary format required the presence of detergents to avoid unreproducible spikes from particle aggregation [13]. Upon application of chips for electrophoresis owing to (i) differences in the supporting material (soda-lime glass instead of fused silica) and (ii) the short separation distance, as well as (iii) the FL labelling of virus particles, electrophoresis of virions in the absence of detergents could be achieved. This was a necessary prerequisite to target processes occurring early in infection by using an in vitro model in which the inner endosomal membrane was mimicked by the lipid membrane of liposomes (vesicles consisting of a lipid bilayer enclosing an aqueous lumen). The possibility (i) to follow the attachment of virions to liposomes [19, 20] and (ii) to detect changes in liposome membrane permeability upon virus uncoating [21, 22] by means of chip electrophoresis have been demonstrated. On the other hand, transfer of the viral RNA genome through this vesicle membrane was assessed via encapsulation of reverse transcriptase in the lumen of liposomes. The released viral RNA served as a template for cDNA synthesis inside vesicles followed by subsequent amplification by PCR and detection via agarose gel electrophoresis [22].

In the current manuscript, we now demonstrate that the combination of molecular beacons (MBs) with chip electrophoresis is also suitable to assess the in vitro release of viral RNA into an aqueous buffer (see Electronic Supplementary Material (ESM) Fig. S1 for a schematic of the study outline). MBs, short, single-stranded oligonucleotides, carry a fluorophore/quencher pair at their respective ends. Under favourable conditions (e.g. the ionic strength, *I*, of a corresponding buffer), sequences at the 3′ and 5′ ends of the MB form a short helix structure (stem) leading to spatial proximity between the fluorophore/quencher pair (closed hairpin) and no FL is recorded. Hybridization of nucleotides not involved in stem formation (i.e. the loop) to a complementary target sequence results in loss of the stem helix structure and hence to an increase in FL (open hairpin) [23].

To date several publications describe the application of MBs for virus genome detection e.g. as FL probes in living cells [24, 25]. Su et al. [26] recently introduced a microfluidic approach to detect fish pathogens via MBs and magnetic beads. In the current manuscript we demonstrate the specific detection of viral RNA by a fast and easy-to-handle chip electrophoresis method. With this we set the stage for a more detailed analysis of the release of the HRV-A2 RNA genome from the protective virus capsid by using MBs. We expect this analytical method to help in obtaining a more detailed view of how the viral RNA genome that is tightly packed inside the viral shell passes through a small orifice in the subviral capsid during infection. The current hypothesis suggests RNA release in the form of a completely unfolded single strand implying that nucleotide sequences along the genome must become available for hybridization in a time-dependent manner from the 3′ towards the 5′ end; by using a set of suitable MBs, such analyses might become possible with the methodology described in the present report.

## Materials and methods

### Chemicals and reagents

Boric acid and DMSO (both pro analysis) were obtained from Fluka (Steinheim, Germany) and sodium hydroxide (≥99 %) from Merck (Darmstadt, Germany). Atto 488, Atto 495 and Atto 633 were from Atto Tec (Siegen, Germany). The three individual fluorophore molecules were dissolved in DMSO (at 2.5, 3.3 and 2.5 mM, respectively) and further diluted as described below. Trolox (6-hydroxy-2,5,7,8-tetramethylchroman-2-carboxylic acid) and Benzonase nuclease (ultrapure grade) were purchased from Sigma Aldrich (Steinheim, Germany). Water (18.2 MΩ cm resistivity at 25 °C) was from a Millipore (Billerica, MA, USA) apparatus.

### Biological material

HRV-A2 was prepared and its infectivity was assessed as described [27]. The 50 % tissue culture infectious dose (TCID_50_/mL) of preparations was typically between 10^10^ and 10^11^ infectious units per mL (refer to ESM Fig. S2 summarizing the data of the virus preparations used). The concentration of virus particles was obtained from nES GEMMA measurements [2, 11, 28] yielding virus peak heights. Comparison of these values to the nES GEMMA peak height obtained for an HRV-A2 preparation whose concentration had previously been measured by CE [27] and taking sample dilution into account enabled us to calculate virus concentrations of all employed batches. It is of note that lipase-treated virus preparations [11] still contained low but detectable amounts of so far unidentified contaminating material. Therefore, it has to be concluded that the purity of HRV-A2 preparations is dependent (i) on the original amount of this contaminating material (prior to lipase digestion) and (ii) on the activity of lipase which differs between enzyme batches. HRV-A2 quality control either via nES GEMMA or TEM [27] thus appears to be necessary to check whether the purity of a given preparation is sufficient for a particular experiment. HRV-B14, another rhinovirus strain, was prepared as above and employed at 2 mg/ml (3.65 × 10^11^ TCID_50_/mL) in 50 mM Tris, pH 7.4 as a negative control for the specificity of the MB.

Oligonucleotides were obtained from Integrated DNA Technologies (Leuven, Belgium). The MB (Atto 633 - tcgcaCAAAA***G***CAAA***T***CA***T***ACG***G***AGGGATGcga - Iowa Black RQ-Sp quencher) targets a region close to the 3′ end of the HRV-A2 genome (nucleotides 7017–7041, see ESM Fig. S3 for the position of complementarity on the viral RNA genome [29]). Uppercase letters are bases complementary to the viral RNA. MB nucleotides were modified with locked nucleotides (LNAs, in bold and italics). Care was exercised to exclude intramolecular recognition of LNAs. An oligonucleotide complementary to the MB (with eight additional nucleotides identical to the viral genome at each end) was employed as positive, single-stranded DNA (ssDNA) control for MB binding. An oligonucleotide of the same length but with a sequence of a different region of the viral RNA was employed as a negative ssDNA control. ssDNA controls did not contain LNA. The MB and controls were purified by HPLC and lyophilised. They were dissolved in 2 mM ammonium acetate, pH 7.2 to yield 0.5 mM stock solutions (based on the amounts specified by the manufacturer). Aliquots were kept at −20 °C. The MB stock solution was subsequently diluted for preparation of samples used in experiments as specified below. It is of note that the MB in the presence of Trolox suffered an alteration over time that impacted on its FL output. The long-term storage of the MB in background electrolyte (BGE) solutions containing Trolox was therefore avoided (unpublished data). When contact of MB with Trolox was limited to a sample preparation step on the day of measurement, no such alteration was observed.

As an additional control to exclude unspecific binding, MB was incubated with RNAs of irrelevant sequence (RNA ladder from Agilent Technologies, Waldbronn, Germany, a mixture of six RNAs of lengths between 0.2 and 6 kb at a total concentration of 150 ng/μL in 0.1 mM EDTA). ESM Fig. S4 depicts the corresponding electropherogram for such a ladder sample obtained from a standard chip gel electrophoresis set-up.

### Instrumentation

Chip electrophoresis was carried out on an Agilent 2100 Bioanalyzer instrument in the presence of electroosmosis employing commercially available, single-use chips [17, 18]. Modifications of the script (software of the instrument) allowed electrophoresis of samples at ambient temperature (typically *T* = 22.5 ± 0.3 °C). Sodium borate, pH 8.5, with and without Trolox as indicated (total ionic strength, *I*, was kept constant at approx. 40 mM) was employed as BGE and sample buffer (SB). Sodium borate was filtered (0.2 μm pore size, surfactant-free cellulose acetate membranes from Sartorius, Göttingen, Germany). Analyte detection was simultaneously at two wavelengths (λ_ex/em_ = 470/525 and 635/685 nm). The instrument optics was adjusted to the position of a respective single-use chip inside the instrument after electrophoretic flushing of the separation channel with 100 nM Atto 633 in BGE. Subsequently, the fluorophore was removed from the separation channel via electrophoresis.

### Sample preparation

Samples contained MB at 40 nM as well as Atto 488 (2.5 μM) and Atto 495 (3.3 μM) as internal standards. Positive control ssDNA target concentrations of up to 400 nM and negative control ssDNA target concentrations of 200 nM were employed as indicated in the respective figures. When RNA ladder was used as negative control, 6 ng/μL was employed (yielding a comparable molar concentration of the RNA and DNA controls). Samples were incubated at 30 °C for 30 min at 800 rpm employing an Eppendorf Thermomixer instrument to check for MB reactivity employing control oligonucleotides. Subsequently, samples were cooled and stored on ice until analysis on the same day. For detection of the RNA released from HRV-A2, the control nucleotides were substituted by HRV-A2. The virus was either directly diluted in BGE or employed after additional removal of low molecular weight components followed by such a dilution step. In both cases, the dilution factor of virus stock was 1:15 (v/v). Removal of low molecular weight sample components was carried out as described elsewhere [30] employing polyethersulfone membrane filters with a 10-kDa molecular weight cutoff (VWR, Vienna, Austria). The final virus concentration was around 80 nM with an infectivity of 2.8 × 10^10^ TCID_50_/mL (overall dilution of HRV-A2 preparation A of 1:16.7 (v/v); for a list of all employed HRV-A2 preparations refer to ESM Fig. S2). Samples were heated for 15 min to 56 °C (800 rpm, Eppendorf Thermomixer) to trigger RNA release. Subsequently, samples were cooled on ice to allow for MB binding and subjected to chip electrophoresis. Negative controls (i) omitted the heating step or (ii) replaced HRV-A2 by HRV-B14, whose RNA sequence has no regions complementary to the MB. (iii) The impact of 15 min heating to 56 °C on MBs was assessed, too.

### Data evaluation

Raw data were exported from the Agilent 2100 Bioanalyzer software. For inter- and intrachip comparability, electropherograms were adjusted with respect to analyte migration according to Reijenga et al. [31] and to the signal heights of the internal standards as detected with the blue LED of the instrument (see “Results and discussion”). The area of the recorded FL signals was obtained by fitting Gaussian-shaped peaks to the data (OriginPro 9.1.0; see ESM Fig. S5 for an exemplary electropherogram).

## Results and discussion

To generate a specific fluorescence signal the closed hairpin of the MB must open, which needs to be triggered via binding of its loop sequence to the complementary sequence of a target. Unfolding without hybridization, which might occur at low *I* or elevated temperature, results in a false positive signal. Therefore, orienting experiments were conducted with various MBs (same stem, different loop) to investigate the dependence of unfolding on *I* of the BGE (not shown). As expected, increase of *I* led to a reduction of the observed electroosmotic flow as well as to a higher proportion of the MB in its closed hairpin conformation, i.e. less FL was recorded. The maximal possible FL gain under the finally chosen *I* conditions was determined via nuclease digestion of the MB, which leads to its complete dequenching. Addition of divalent cations (e.g. Mg^2+^) known to stabilize the MB stem was omitted as these had been found to stabilize viral uncoating intermediates [9].

### Chip electrophoresis of MBs

We carried out chip CE measurements in sodium borate, *I* = 40 mM (pH 8.5). For intra- and interchip comparison of the electropherograms, we included two additional fluorophores in samples similar to Bidulock and colleagues, who used caesium and lithium chloride as internal standards in sodium chloride analysis on home-made chips [32]. In our case, Atto 488 and Atto 495 gave rise to FL signals detectable with the blue LED of the applied instrument and did not interfere with analyte signals detected at λ_ex/em_ = 635/685 nm (see ESM Fig. S6A). In the specified BGE, Atto 495 is zwitterionic and migrates with the electroosmotic flow (EOF), whereas Atto 488 is negatively charged (the structure of dyes is available from the homepage of the manufacturing company). Migration of both peaks of the internal standards is such that electropherogram normalization, as described by Reijenga and colleagues [31], is feasible and slight changes in the EOF can be corrected. As all samples included the same amount of both dyes, we were also able to correct for slight changes in FL intensities. An intensity correction factor for each of the two internal FL standards was calculated separately and the average of these two numbers was employed to finally correct the FL intensity of a corresponding electropherogram. ESM Figs. S6B and C demonstrate the utility of this approach for correction of the blue traces of several electropherograms obtained from different chips. Upon normalization of both axes the signals were comparable.

Although we expected a completely closed state of the MBs under the given electrophoresis conditions and in the absence of a target molecule, our analyses showed that the MB exhibited residual FL in the absence of cognate oligonucleotides or viral RNA targets (ESM Fig. S7). However, compared to the high signal obtained after digestion with Benzonase this background was found to be negligible (an approx. 25-fold increase of FL was recorded upon Benzonase treatment). Therefore, we were indeed able to set the stage for our intended in vitro viral RNA release detection method by slight advancements of chip CE in comparison to our originally published set-up [17, 18] i.e. by inclusion of two internal standards as well as by choosing conditions where the majority of the MB is in its closed hairpin state (i.e. by adjustment of *I* of the BGE). Additionally, we modified the software of the instrument (script) to allow electrophoretic separations at ambient temperature (*T* = 22.5 ± 0.3 °C).

### Impact of BGE additives on FL response of MB

**Fig. 1:**
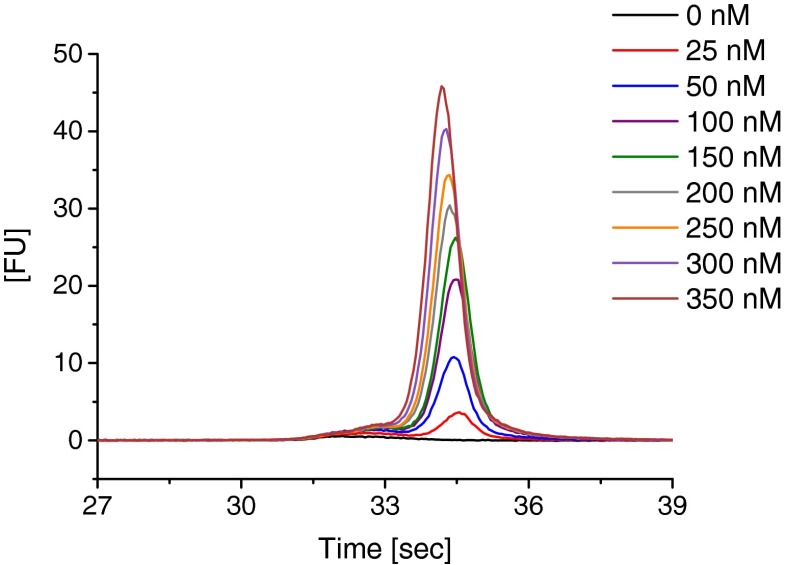
Chip CE results for MB binding to positive control ssDNA oligonucleotides employed at indicated concentrations. With increasing concentrations of the control oligonucleotide an increase of the FL signal is observed in the electropherograms. Sodium borate, pH 8.5 (*I* = 40 mM) without any additives was employed as SB and BGE. Data was aligned as described in the text

Subsequent experiments targeted the MB’s reactivity in the presence of the respective positive control ssDNA oligonucleotide. With increasing concentration of this oligonucleotide an increase of FL was recorded (Fig. 1). No such increase was detected with the negative control oligonucleotide or with the RNA ladder (ESM Fig. S8A, B).

**Fig. 2:**
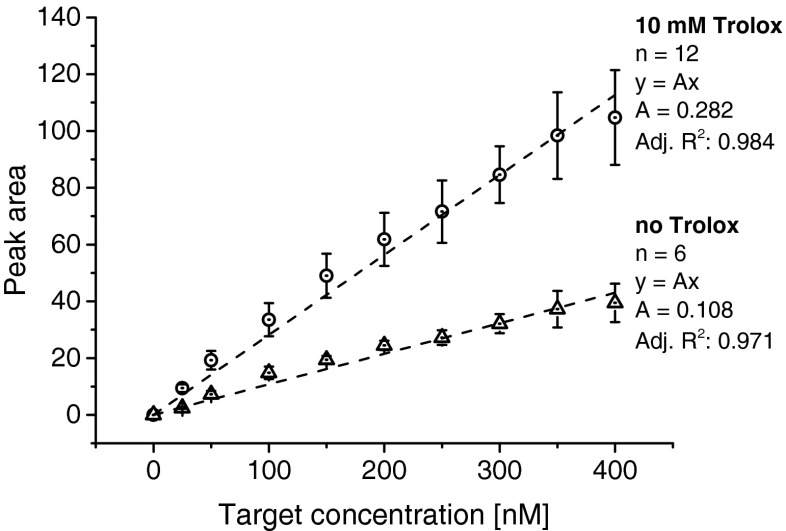
Addition of 10 mM Trolox to the BGE (*I* constant at 40 mM) resulted in a two- to threefold increase of FL signals obtained for the MB/positive control ssDNA oligonucleotide complex as a result of reduced photobleaching and blinking. Values of symmetric Gaussian peaks fitted to electropherograms were taken for calculation. The number of repeats per data point is indicated in the figure, standard deviation values are shown. Data points are approximated via linear fits

Several compounds reduce blinking and photobleaching of fluorophores [33] in FL microscopy. In our case Trolox (6-hydroxy-2,5,7,8-tetramethylchroman-2-carboxylic acid) at 10 mM concentration in the BGE and SB increased the FL signal by approximately two- to threefold (Fig. 2). Fitting Gaussians to the electropherogram peaks yielded a linear correlation between the concentration of the positive control oligonucleotide and the area of the FL peak. In the presence of 10 mM Trolox, the FL signal equalled 0.282 times the concentration of the positive control oligonucleotide (in nanomoles per litre). In the absence of Trolox the slope of the linear correlation dropped to 0.108. Higher concentrations were not tested because of the limited solubility of Trolox (around 3 mg/mL which corresponds to approx. 12 mM in phosphate buffered saline, pH 7.2; www.scbt.com/datasheet-200810-trolox.html). Additive concentrations close to its solubility limit might lead to clogging of the chip channels. Use of ascorbic acid as an alternative additive (up to 6 mM) failed to increase the FL yield (data not shown).

### Detecting RNA release via MBs and chip electrophoresis

**Fig. 3:**
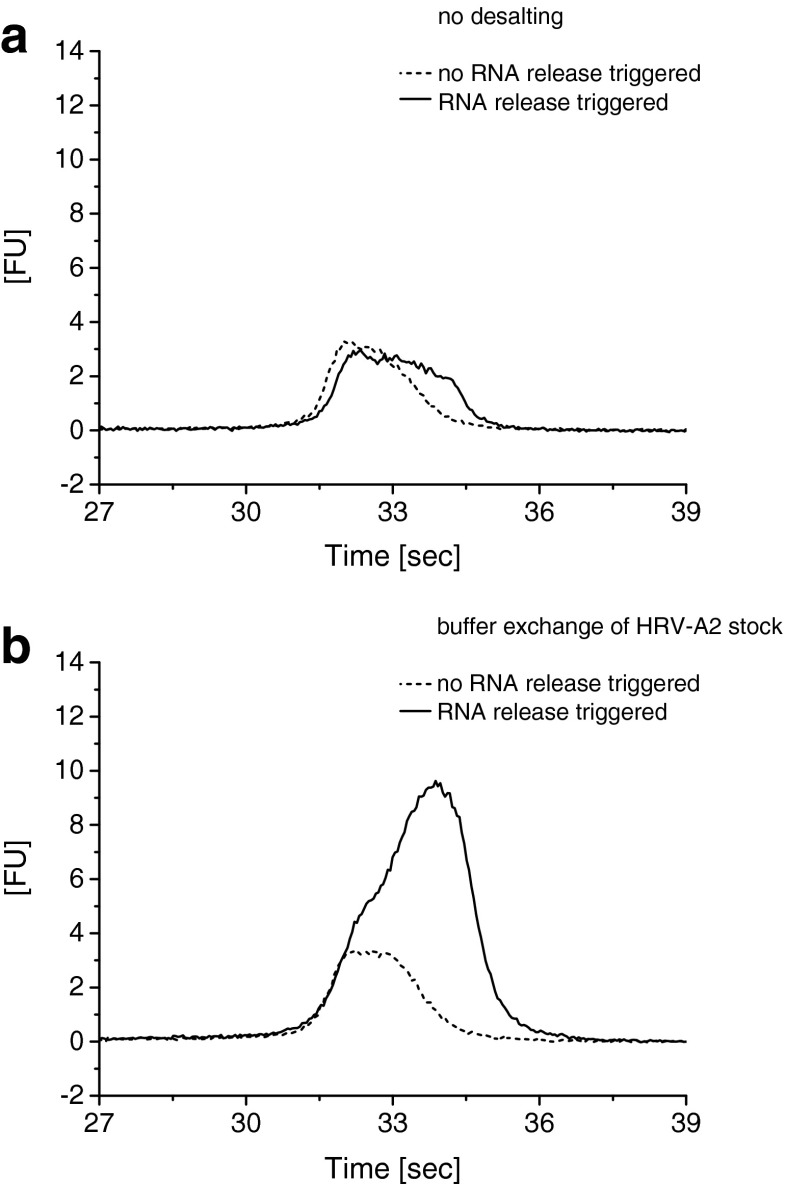
Detection of free viral RNA by using our chip CE set-up. RNA release was triggered through heating of the virus preparation (15 min to 56 °C). As a negative control, the sample was kept in ice and the viral RNA remains inside the protein shell and inaccessible to the MB. Buffer exchange of the HRV-A2 batch was found to strongly increase the signal of the viral RNA (compare **a**, which is the result without buffer exchange, and **b**, which is the result including a sample buffer exchange step). Sodium borate, pH 8.5 including 10 mM Trolox (*I* = 40 mM) was employed as SB and BGE. Data was aligned as described in the text

Following our experiments with control oligonucleotides we mixed MB with HRV-A2, triggered virus uncoating via heating for 15 min to 56 °C, and carried out chip CE as above. Desalting of HRV-A2 preparations was beneficial for the FL signal obtained from MB binding the viral RNA possibly because uncoating might have been reduced owing to the stabilizing effect of Mg^2+^ and other divalent cations present in the HRV-A2 stock solution [9]. After this buffer exchange we observed clear and specific signals for the MB–RNA complex (compare Fig. 3a without HRV-A2 desalting prior virus uncoating to Fig. 3b where HRV-A2 desalting had been carried out before initiating viral RNA release at 56 °C). The test with MB alone (in the absence of virus) showed only a low response to sample heating to 56 °C indicating that it folded back on cooling (ESM Fig. S8C). Likewise, no reactivity of the MB with RNA from HRV-B14 was recorded indicating that the obtained MB/RNA signal was specific (ESM Fig. S8D). RNase activity after release of the viral genome slightly impacted the obtained MB–RNA complex signal. Given samples were stored on ice as well as under light protection and measurements were carried out within a few hours after sample preparation areas recorded for the complex were found to be within approx. 10 % of an average (ESM Fig. S9).

### Challenges of the experimental set-up

**Fig. 4:**
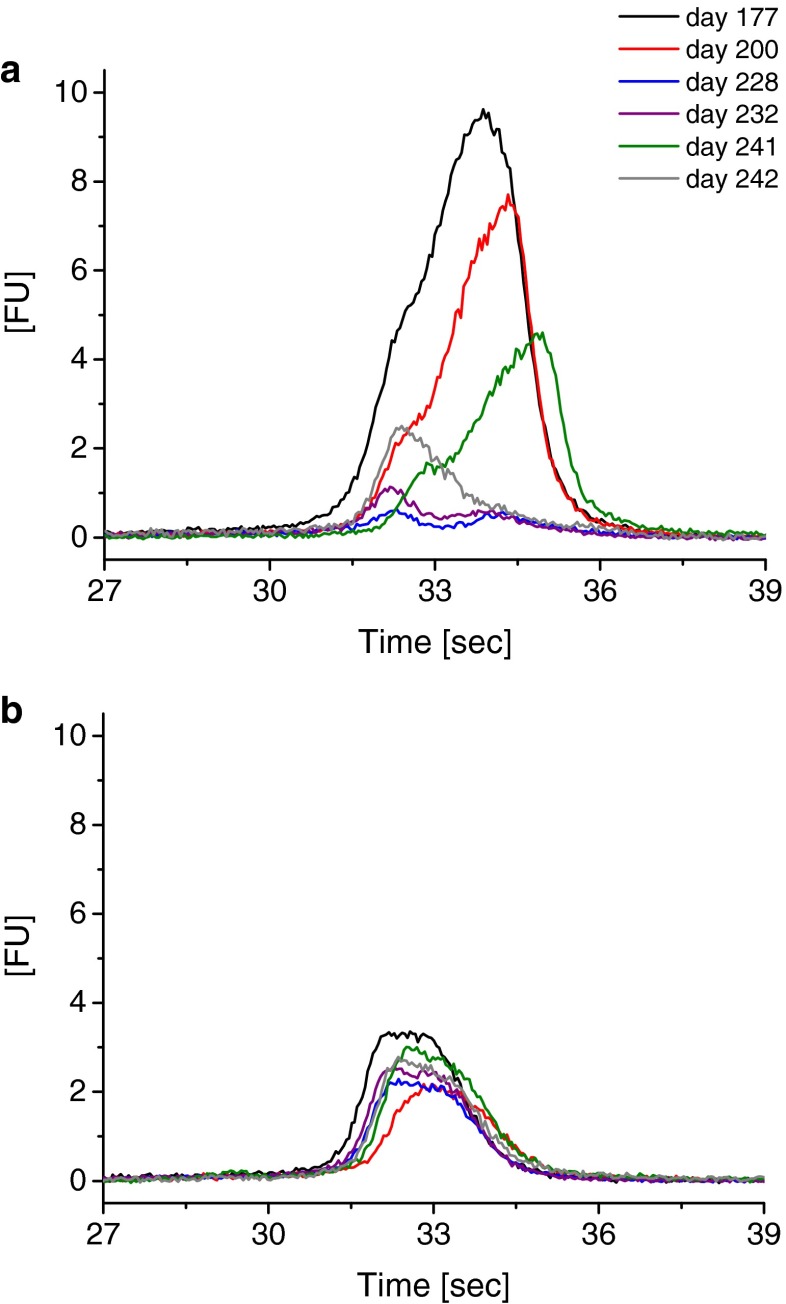
Decline of the MB/RNA signal in the course of chip CE experiments over time. Viral RNA release experiments as presented in Fig. 3 were repeated at the indicated times (days) after preparation of the original HRV-A2 batch. Samples were prepared freshly from an HRV-A2 stock for each time point. A decline in the FL signal for the MB/RNA signal (**a**) was observed over time which was ascribed to degradation of the viral RNA genome. No significant decline is observed for the negative control electropherograms for the MB in the presence of HRV-A2 but without triggering of viral RNA release (**b**)

Having demonstrated the applicability of our set-up to qualitatively follow the release of HRV-A2 RNA employing an MB and chip CE, we were interested in the quantification of the obtained FL signals. However, measurements of identically prepared samples showed a significant decline of the MB–RNA signal over time of storage at −70 °C and the numbers of freezing and thawing cycles of the virus stock solution (Fig. 4a; note that samples were prepared freshly for each experiment). It is important to mention that a decline of infectivity by as much as one log TCID_50_/mL even during storage at −80 °C for several weeks was frequently observed. However, fluorescence of the MB measured on incubation with native HRV-A2 whose RNA is inaccessible (Fig. 4b) did not alter in FL over time. This makes it unlikely (i) that the MB interacts with virus and/or virus aggregates, which might reduce the concentration of free MB in solution and (ii) that the MB is degraded. Instead we assume that repeated freezing/thawing of the virus stock solution impacted on the signal as a result of the viral RNA being degraded.

Breathing, i.e. transient exposure of capsid-internal N-terminal sequences, of VP1 and VP4 was reported for picornaviruses [15] and similar dynamics of virus capsid components have been observed for hepatitis B virus capsids [34]. These transient rearrangements of the capsid proteins might account for the RNA becoming accessible for RNases, especially during freezing and thawing. In this context it is of note that Gauntt had observed fragmentation of the RNA in virions of another serotype [35].

Material apparently released from HRV-A2 later in the experimental series (i.e. after virus storage at −70 °C and repeated freezing/thawing leading to viral RNA degradation) resulted in quenching of overall FL. Hence, special care has to be exercised with virus storage and freezing/thawing. Also, inclusion of RNase inhibitors in samples and stocks might be beneficial to avoid eventual degradation of the viral RNA via RNases that might gain access during temporary opening of pores given that an impact of these additional components on chip CE can be excluded.

On the basis of the linear correlation between the concentration of the control oligonucleotide and FL, we calculated the amount of viral RNA that was released on heating. This yielded 43 nM for the most prominent MB–RNA complex signal (at day 177 after HRV-A2 preparation; Fig. 4a). When the virus was then frozen and thawed the RNA concentration was found to be decreased, which is in line with the well-known natural decay of viruses.

**Fig. 5:**
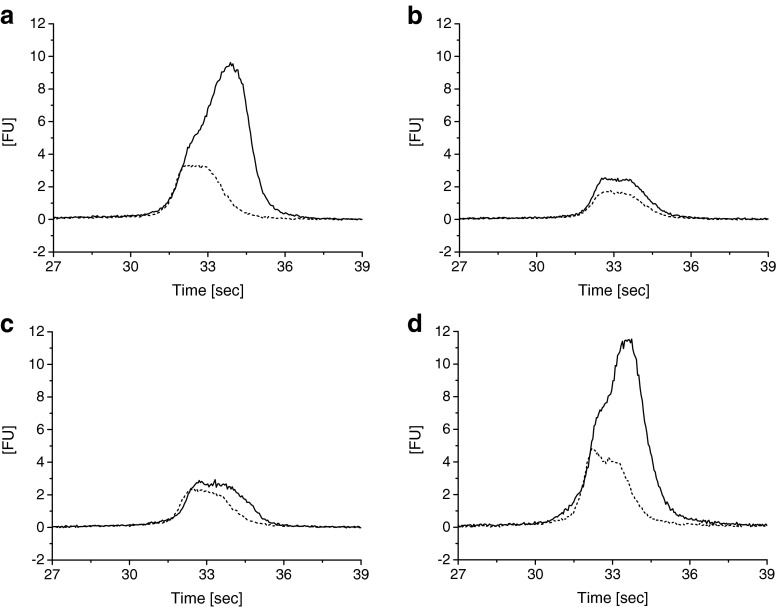
Chip CE results for different HRV-A2 batches **a**–**d**. (see ESM Fig. S2). Virus stock solutions were subjected to buffer exchange. Depending on the quality of the preparation (purity and infectivity), a signal for the MB–RNA complex is obtained. Conditions as noted for Fig. 3. No RNA release triggered via sample heating, *dashed line*; RNA release triggered by sample heating for 15 min to 56 °C, *solid line*

Analysis of HRV-A2 preparations other than A led to similar results (see ESM Fig. S2 for an overview of virus preparations) when the TCID_50_/mL values and purities were comparable, as in preparation D. In the case of low virus concentrations (e.g. batch B, see ESM S1) or highly impure virus preparations (e.g. batch C, see ESM S1), no MB–RNA complex could be detected (Fig. 5a–d for indicated HRV-A2 preparations). Batch C showed considerable amounts of the previously described contaminant as well as a protein impurity, possibly ferritin, as inferred from gas-phase electrophoresis (nES GEMMA).

## Conclusions

The major aim of this study was the development of a fast, sensitive and easy-to-handle analytical method to detect viral RNA by using an MB and chip CE to set the stage for further investigation of the mode of release of the RNA genome of a human rhinovirus. Intra- and interchip comparison of results by using internal standards was good (analytes were detected with the second wavelength of the instrument without interference from internal standard signals). Addition of Trolox to the BGE increased the FL signals substantially. Our results demonstrate that the release of the viral RNA genome from the viral capsid can indeed be monitored via the combination of an MB and chip CE. However, during our investigations we noted a significant decrease of the signal measured with the MB over time of storage at −70 °C and repeated freezing/thawing, which we attributed to degradation of the viral RNA genome. Nevertheless, given that an HRV-A2 preparation was of sufficiently high purity and the virus concentration was in the range of at least 10^10^ TCID_50_ in the final sample, results between preparation batches were very similar. Future work might substantially lower this detection threshold and will also allow the extension of our presented in vitro set-up to the analysis of other viruses.

## Electronic supplementary material

Below is the link to the electronic supplementary material.

ESM 1 (PDF 797 KB)
