# Supplementary material for: In vitro RNA release from a human rhinovirus monitored by means of a molecular beacon and chip electrophoresis
Source: Anal Bioanal Chem. 2016 Mar 28;408:4209–17. doi: 10.1007/s00216-016-9459-2 (PMC4875947; doi:10.1007/s00216-016-9459-2)
Supplement: Supplementary file 1 — (PDF 797 KB) [file 216_2016_9459_MOESM1_ESM.pdf]

Analytical and Bioanalytical Chemistry

Electronic Supplementary Material

In vitro RNA release from a human rhinovirus monitored by means of a molecular beacon and chip electrophoresis

Victor U. Weiss, Christina Bliem, Irene Gösler, Sofiya Fedosyuk, Martin Kratzmeier,
Dieter Blaas, Günter Allmaier

The supplementary part of the manuscript gives additional information on:

(i) the study principle (S1),

(ii) HRV-A2 preparation batches (S2),

(iii) position of complementary to the MB on the HRV-A2 RNA genome (S3),

(iv) the RNA ladder including six RNA transcripts of various length used as negative control (S4); a chip gel electropherogram (Agilent 2100 Bioanalyzer RNA Nano kit) is presented,

(v) date evaluation of FL signals obtained upon MB binding to positive control ssDNA oligonucleotides (S5),

(vi) the concept of electropherogram alignment (S6),

(vii) the FL increase obtained upon MB digestion via the nuclease benzonase (S7),

(viii) signals obtained for negative controls (S8) including ssDNA (A), RNA ladder (B), MB heated for 15 min to 56°C (C) and HRV-B14 (D),

(ix) the impact of RNase digestion over time on MB / RNA complexes after triggering of viral RNA release from protective capsids (S9); samples were stored on ice and under light protection after RNA genome release; between first (left) and last (right) measurement lay approx. 4 hours.

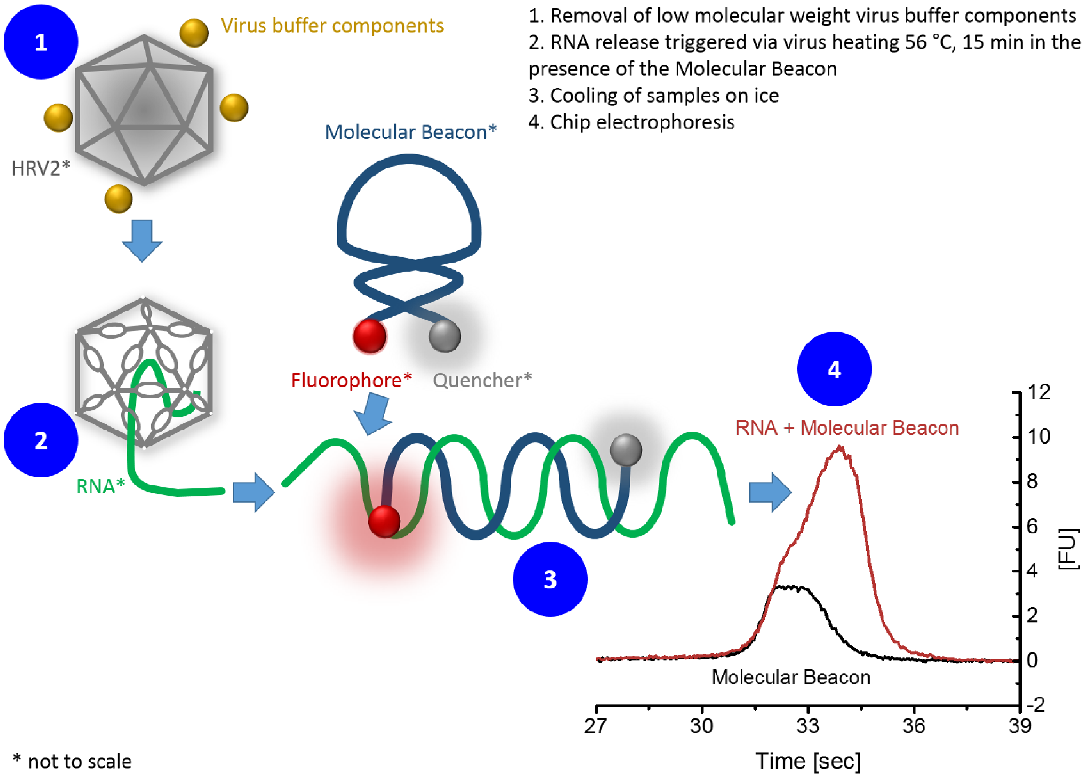

Supplementary Figure S1: Schematic of the study principle

<table>
<tr><th>HRV-A2:
Preparation date
Preparation mode</th><th>Preparation ID</th><th>$TCID_{50}$/mL</th><th>Virus particles [mg/mL]</th><th>nES GEMMA spectra</th></tr>
<tr><td>03.02.2014
2nd fraction
Standard preparation with additional lipase digestion [Weiss, V. U. et al, 2015 Analytical Chemistry]</td><td>#A</td><td>$4.64 \cdot 10^{11}$</td><td>11.9</td><td>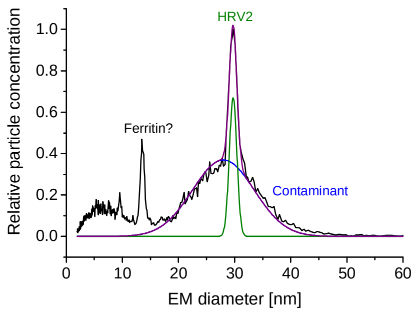
</td></tr>
<tr><td>03.02.2014
1st fraction
Standard preparation with additional lipase digestion [Weiss, V. U. et al, 2015 Analytical Chemistry]</td><td>#B</td><td>$2.02 \cdot 10^{10}$</td><td>n.d.</td><td>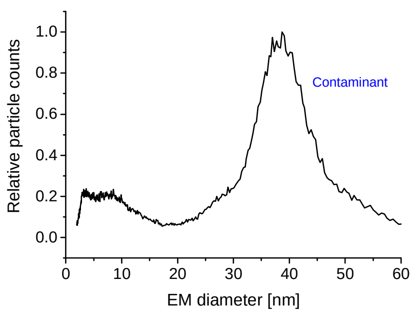
</td></tr>
<tr><td>04.07.2014
2nd fraction
Standard preparation with additional lipase digestion [Weiss, V. U. et al, 2015 Analytical Chemistry]</td><td>#C</td><td>$5.62 \cdot 10^{10}$</td><td>0.3</td><td>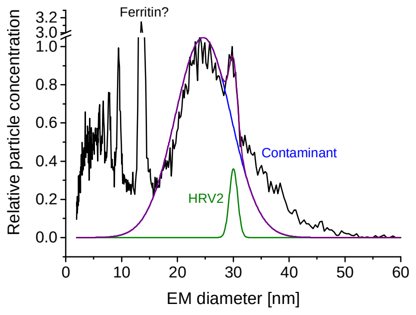
</td></tr>
<tr><td>30.01.2015
1st fraction
Standard preparation with additional PEG precipitation step prior sucrose density centrifugation</td><td>#D</td><td>$4.64 \cdot 10^{11}$</td><td>4.7</td><td>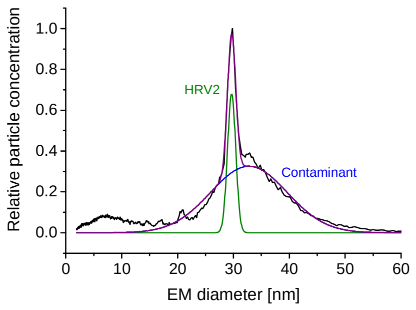
</td></tr>
</table>

Supplementary Figure S2: Overview on HRV-A2 preparation batches; n.d. value not determinable

**(5’)VP_ttaaaactggatccaggttgttcccacctggatttcccacagggagtggtactctgttattacggtaactttgtacgccagttttatctcccttcccccatgtaacttagaagtttttcacaaagac
caatagccggtaatcagccagattactgaaggtcaagcacttctgtttccccggtcaatgttgatatgctccaacagggcaaaaacaactgcgatcgttaaccgcaaagcgcctacgcaaagcttagta
gcatctttgaaatcgtttggctggtcgatccgccatttcccctggtagacctggcagatgaggctagaaataccccactggcgacagtgttctagcctgcgtggctgcctgcacaccctatgggtgtgaag
ccaaacaatggacaaggtgtgaagagccccgtgtgctcgctttgagtcctccggcccctgaatgtggctaaccttaaccctgcagctagagcacgtaacccaatgtgtatctagtcgtaatgagcaattg
cgggatgggaccaactactttgggtgtccgtgtttcactttttcctttatatttgcttatggtgacaatatatacaatatatatattggcaccatgggtgcacaggtttcaagacaaaatgttggaactcact
ccacgcaaaactctgtatcaaatgggtctagtttaaattattttaacatcaattatttcaaagatgctgcttcaaatggtgcatcaaaactggaattcacacaagatcctagtaaatttactgacccagtt
aaggatgttttggaaaagggaataccaacactacagtcccccacagtggaggcttgtggatactctgataggattatacagattaccagaggagattcaaccataacctcacaagatgtggctaatgc
tatcgttgcgtatggtgtttggccacattatctatcctccaaggatgcctctgcaattgataaaccctctcaaccagatacatcttctaatagattttatactctaaggagtgtgacctggagcagttcctca
aagggttggtggtggaaactacctgatgcactcaaggacatgggtatttttggtgaaaacatgttttatcattacctgggtaggagtggatacacaatacatgtgcagtgtaatgctagtaaatttcacc
agggtacactaattgttgctctgatacctgagcatcagattgcaagtgccttacatggcaatgtgaatgttggttacaactacacacacccaggtgaaacaggcagggaagttaaagctgagacgaga
ttgaatcctgatctacaacctactgaagagtattggctaaactttgatgggacactccttggaaatattaccatattccctcatcaatttatcaacttgaggagtaataattctgccacaataattgcccct
tatgtcaatgcagttcctatggattcaatgcggagccacaataattggagtttggtaataataccaatatgtccccttgagacatcaagtgcaattaacacaatacctattacaatatctataagccccat
gtgtgcagagttttccggcgcgcgtgccaagcgtcaaggattaccagttttcatcacaccaggttcaggacagtttttgacaacagatgatttccaatccccatgtgcacttccctggtatcacccaactaa
ggaaatttctattccaggtgaggttaaaaatttggttgaaatttgtcaagtagacagcctagtaccaataaataacactgacacctacatcaatagtgaaaatatgtattctgttgtattgcaatcatca
attaatgcaccagataagatcttctctattcgaacagatgttgcttcccaacctttagctactactttgattggtgagatatctagctatttcacccactggacagggagtctccgtttcagcttcatgttttg
tggtactgccaacactactgttaagcttttgttggcatacacaccacctggtatcgcagaacccaccacaagaaaggatgcaatgctaggcactcatgttatatgggatgtggggttgcagtctacaata
tcaatggtagtgccatggattagcgctagtcattatagaaacacatcaccaggtagatctacatctgggtacataacatgctggtatcagactagattagtcattccacctcagaccccaccaacagcta
gattgttatgttttgtatctgggtgcaaagacttttgcttgcgcatggcacgagatactaacctacacctgcaaagtggtgcaatagcacagaaccctgttgagaattatatagatgaagttcttaatgaa
gttttagttgtcccaaatattaatagtagtaaccccacaacatcaaattctgccccagcattagatgctgcagaaacagggcacactagtagtgttcaaccagaggatgtcattgaaactaggtatgtgc
agacatcacaaacaagagatgaaatgagtttagagagttttcttggcagatcaggatgcatacatgaatctaaattagaggttacacttgcaaattataacaaggagaattttacagtgtgggctatta
atctacaagaaatggctcaaattagaaggaaatttgaattgttcacctatactaggtttgattctgaaataaccctagttccatgcatttccgcccttagtcaggacattggacacatcacaatgcaatac
atgtatgttccaccaggtgcaccggtgcccaatagtagggacgattatgcatggcagtctggcactaatgcctctgttttctggcaacatggacaggcttatccaagattttccttacctttcctaagtgtg
gcatctgcttattacatgttttatgatgggtatgatgaacaagatcaaaactatggtacagcaaacacaaataacatggggtcactatgctctaggatagtaacagagaaacacattcataaagtacat
ataatgacaagaatctatcacaaggctaaacatgtcaaggcatggtgtccacgcccacccagagcgcttgagtatactcgtgctcatcgcactaattttaaaattgaggataggagtattcagacagca
attgtgaccagaccaattatcactacagctggccccagtgacatgtatgttcatgtaggtaaccttatttatagaaatcttcatcttttcaactctgagatgcatgaatctattttggtatcttattcatcaga
tttaatcatttaccgaacaaacactgtaggtgatgattacattccctcttgtgattgtacccaagctacttattattgcaaacataaaaatagatacttcccaattacagttacaagccatgactggtatga
aatacaggaaagtgagtactatcccaaacacatacagtacaatttgttgattggtgagggcccttgtgaaccaggtgactgtggtggaaagttgctatgcaaacatggtgtcataggtatagtaacagc
tggtggtgataatcatgtggcttttattgaccttagacacttccattgtgctgaagaacaaggggttacagattatatacatatgctaggagaagcatttggaaatggatttgtggatagtgtaaaagaa
catatacatgccataaacccagtaggaaatatcagcaagaaaattattaaatggatgttgagaataatatcagcaatggtcataataattagaaactcttctgacccccaaactatattagcaacactc
acactgattgggtgttctggatcaccctggagatttttaaaggaaaaattctgtaaatggacacagcttaattatatacacaaagaatcagattcatggttaaagaaatttactgaagcatgcaatgca
gctagagggcttgaatggatagggaataagatatctaaatttattgaatggatgaagtcgatgctcccgcaagctcaattgaaggttaagtacttaaacgagcttaaaaaactcaacctatacgaaaa
gcaagttgagagcttgcgggtggctgacatgaaaacacaagaaaaaattaaaatggaaatagacactttacatgatttgtcacgtaaatttctacctttgtatgcaagtgaggcaaaaaggataaaa
accctatacattaaatgtgataatatcatcaagcagaagaaaagatgtgaaccagtagctatagttattcatggaccacctggtgctggcaaatctataacaacaaatttcctggccaaaatgataact
aatgatagtgacatatactctctacctcctgatccaaaatattttgatggttatgaccaacagagtgtagtaataatggatgacattatgcagaatccagccggggatgacatgacactgttctgccaaa
tggtttctagtgttacatttataccaccaatggctgatctaccagataaaggcaaggcttttgattctaggtttgtattatgcagcacaaatcattcccttctaacacccccgacaataacttcactacctgc
aatgaatagaagatttttcctagatttagatataatagtacatgataacttcaaagatccacagggcaaacttaatgtggcagcagcgtttcgaccatgtgatgtagataatagaataggaaatgcacg
ttgttgtccatttgtgtgtggaaaagcagtttctttcaaagatcgtaactcttgcaacaaatacagccttgcgcaggtgtacaacataatgattgaagaagacagacggagaagacaagtggttgatgtc
atgacagctatattccaagggccaattgatatgaaaaacccaccaccacctgctattactgacttgctccagtctgttagaacccctgaagttattaagtattgtgagggtaatagatggataattccag
cagaatgcaagatagaaaaggagttgaacttggctaacacaatcataacaatcattgcaaatgttattggtatggcgagaataatatatgttatttacaaactttttttgcacattacagggaccatattc
aggagaaccaaagcccaagactaaaatcccagaaaggcgtgtagtaacacagggaccagaggaggaatttgggatgtctttaattaaacataactcatgtgttattacaacagaaaatgggaaatt
cacaggtcttggagtatacgacagatttgtggtcgtaccaacacatgcagatcctggaaaggaaattcaggttgatggtataactacaaaagtcattgactcatatgacctatacaacaagaatgggat
aaagctagaaataacagtacttaaattagatagaaatgaaaaatttagagatatcaggagatatatacctaacaatgaagatgattaccccaattgcaacttagcactgctagcaaaccagcctgaa
ccaactataatcaatgttggagatgttgtatcctatggcaatatactgctcagtggcaaccaaacggctagaatgcttaaatacagttacccaactaaatctggttactgtggaggtgtcttatacaaaa
ttgggcaagtgcttggaatacatgttgggggcaatggtagggatggtttctcagctatgttactcagatcctatttcactgatgttcagggccaaataacgttatcaaagaagaccagtgaatgtaacct
acccagtatacacaccccatgcaaaaccaaattgcagcctagtgttttctatgatgtattccctggttcaaaagaaccagctgtgttgtctgaaaaagatgcccggttacaagttgatttcaatgaagca
ctattttctaaatacaaagggaatacagattgctccattaatgaccacataagaattgcatcatcacattatgcagcacaactcattaccttagatattgacccaaaacctattacacttgaggacagtg
tctttggcactgatggattagaggctcttgatttgaacactagcgcaggatttccatatattgcaatgggagttaaaaagagagatttaataaacaacaagaccaaggatataagcaaacttaaagaa
gcaattgacaaatacggagttgacttacctatggtcaccttcttgaaagatgaactcagaaagcatgaaaaggtaattaaaggtaaaactagagttattgaagctagtagtgtgaatgataccctatta
tttagaacaacttttggcaacctcttttcaaagttccacttgaatcctggaattgttactggatcagcagttggatgtgatccagaggtgttttggtcaaaaataccagcaatgttggatgataaatgtatt
atggcttttgattatacaaattatgatggtagtatacaccctatttggtttgaagctcttaaacaggtactggtagatctatcatttaatccaacattaatagatagactatgcaagtctaaacacatcttc
aaaaatacatactatgaagtggagggaggtgtaccatctgggtgttcaggtactagtatttttaacactatgatcaataatattatcataaggaccttagtgttagatgcatacaagaatatagatctag
ataagcttaagataattgcctatggtgatgatgtcatattctcatacatacatgaactggacatggaggctatagcaatagagggtgttaaatatggtttgactataactcctgctgataaatctaacac
atttgtaaaattagactatagcaatgttactttttaaaaagagggtttaagcaagatgagaagtataactttctaatacatccaactttccctgaagatgaaatatttgaatccatcagatggacaaag
aaaccatcacaaatgcatgaacatgtgttgtctctgtgtcacttaatgtggcacaatggacgtgacgcatacaaaaaatttgtggagaagatacgcagtgtaagcgctggtcgtgcactgtacatccct
ccgtatgatttgcttttgcatgagtggtatgaaaaattttaaagatat8agaaatagtaaactgatagtttattagttttataaaaaaaaaaaaaaaaaaaaaaaaaaaaaaaaaaaaaaaaaaa
aaaaaaaaaaaaaaaaaaaaaaaaaaaaaa (3’)**

Supplementary Figure S3: The position of complementary to the MB on HRV-A2 RNA [29] is marked. Additionally, the position of the poly-A tail at the 3’ end is indicated; note that this poly-(A) tail is between 70 and 150 bases long.

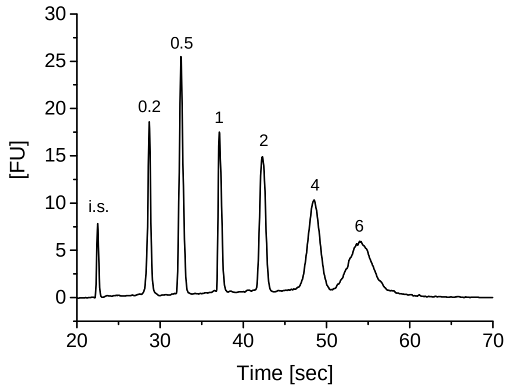

Supplementary Figure S4: Chip gel electropherogram (Agilent 2100 Bioanalyzer RNA Nano Kit) of a RNA ladder with six transcripts of the indicated lengths (in kbases). Note that gel-filled channels were used in this analysis. i.s. internal standard

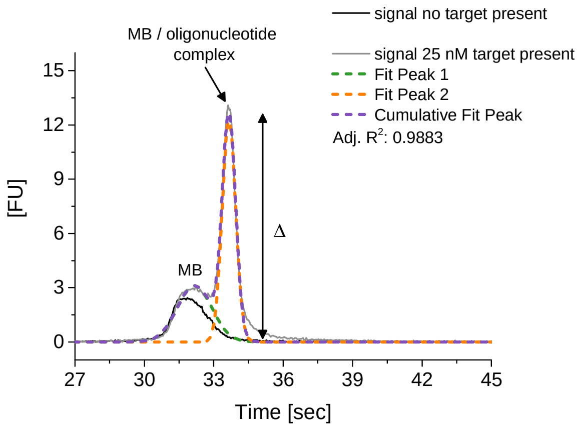

Supplementary Figure S5: Exemplary electropherograms to demonstrate data evaluation of FL signals obtained for the MB complexed to positive control ssDNA oligonucleotides (25 nM target concentration). Gaussion shaped peaks were fitted to data (OriginPro 9.1.0). An increase in FL upon complex formation with the complementary target is indicated by ‘Δ’. Area values of fitted peaks are taken for calculation.

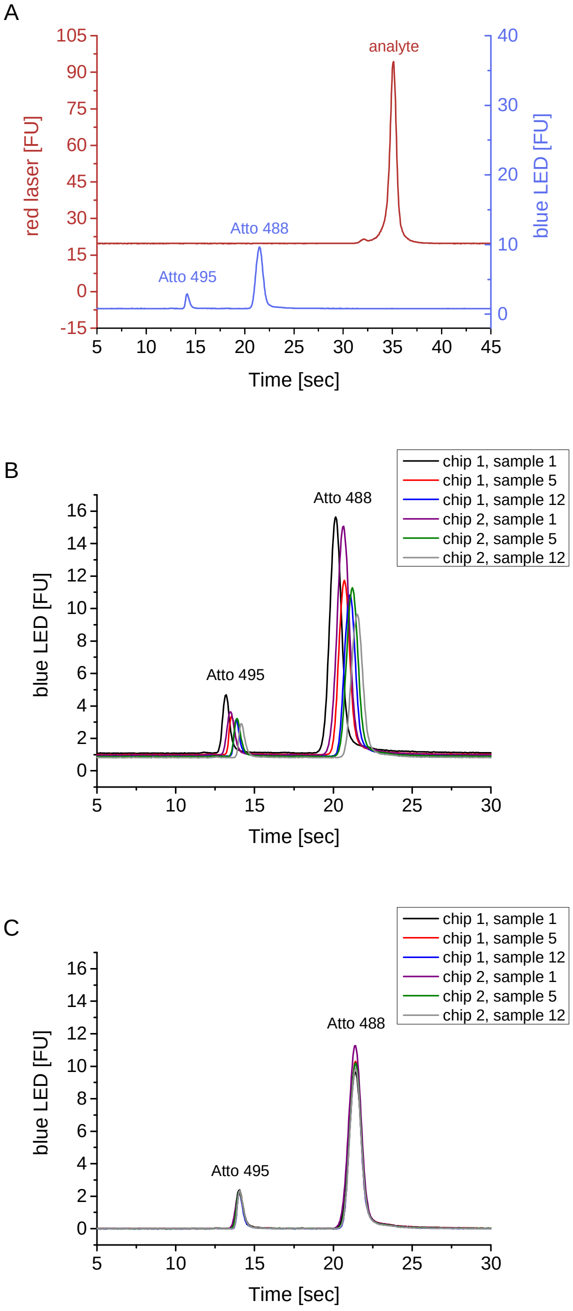

Supplementary Figure S6: Chip CE on the Agilent 2100 Bioanalyzer offers the possibility of simultaneous analyte detection at two wavelengths, $\lambda_{ex/em}$ = 470 / 525 nm (blue LED) and 635 / 685 nm (red laser), respectively (A). The blue trace of the instrument is employed for the analysis of internal standards (Atto 495 and Atto 488) spiked to samples. Inter- and intrachip variations in analyte migration and sensitivity (B) are compensated according to Reijenga et al. (x-axis) [31] and according to the height of FL signals (y-axis), (C).

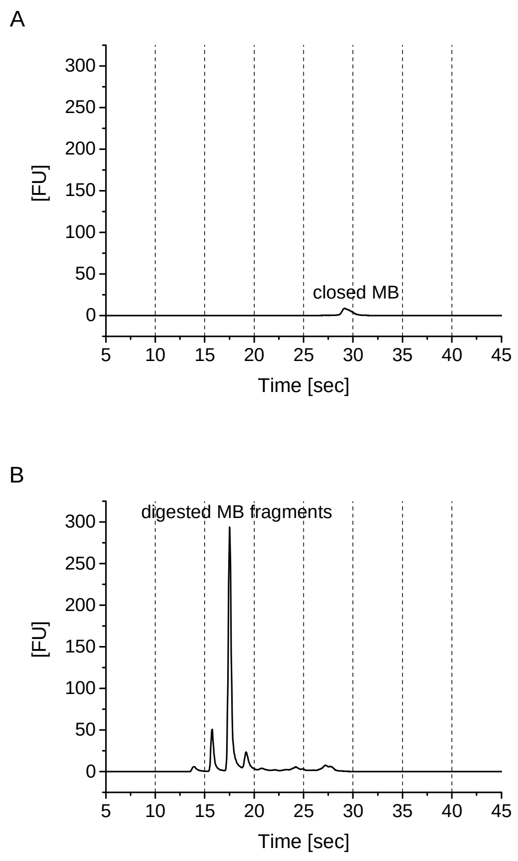

Supplementary Figure S7: Chip CE data of the MB under conditions (sodium borate, pH 8.5, ionic strength of 40 mM) preferably yielding closed probes (A) and after digestion of molecules with benzonase (B) indicating the maximum possible increase in FL. It is of note that the MB concentration (other than for subsequent measurements) was at 100 nM. Digestion was for 2 hours at 30°C and 800 rpm on an Eppendorf Thermomixer employing 0.6 units enzyme in the sample.

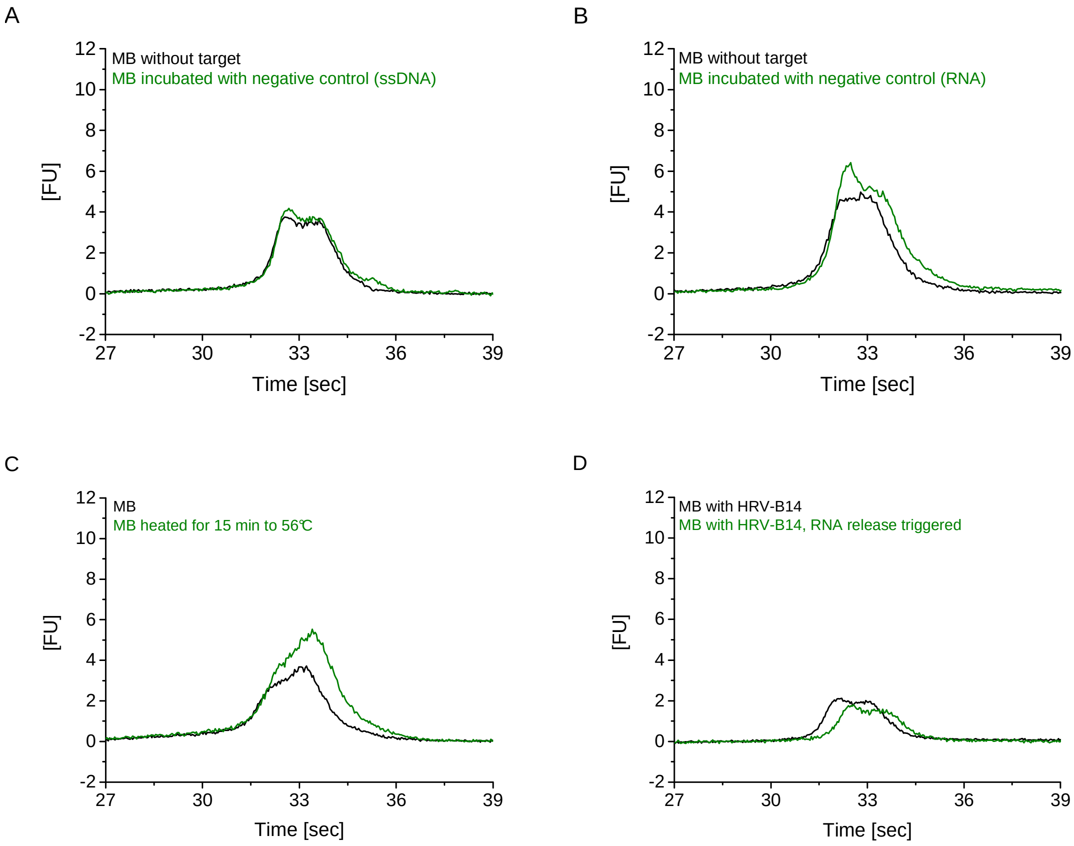

Supplementary Figure S8: Negative controls demonstrating no MB interaction with the negative ssDNA control (A) or the RNA ladder (B) as well as no significant signal increase for the MB alone upon sample heating for 15 min to 56°C followed by cooling (C) or incubation with another HRV strain (HRV-B14) and heating for 15 min to 56°C followed by cooling (D).

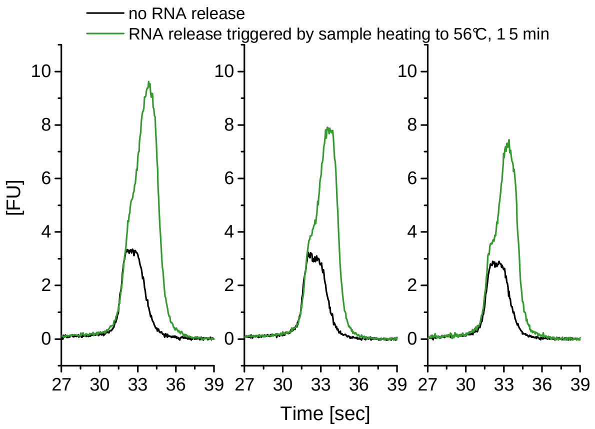

Supplementary Figure S9: In case RNA release and sample measurement were within several hours and sample storage was on ice and under light protection the obtained signals for the MB / RNA complex were in the range of approx. 10 % from an average - between first (left) and last (right) measurement lay approx. 4 hours.
